# COVID-19 – an opportunity to improve access to primary care through organizational innovations? A qualitative multiple case study in Quebec and Nova Scotia (Canada)

**DOI:** 10.1186/s12913-022-08140-w

**Published:** 2022-06-08

**Authors:** Mylaine Breton, Emily Gard Marshall, Véronique Deslauriers, Mélanie Ann Smithman, Lauren R. Moritz, Richard Buote, Bobbi Morrison, Erin K. Christian, Madeleine McKay, Katherine Stringer, Claire Godard-Sebillotte, Nadia Sourial, Maude Laberge, Adrian MacKenzie, Jennifer E. Isenor, Arnaud Duhoux, Rachelle Ashcroft, Maria Mathews, Benoit Cossette, Catherine Hudon, Beth McDougall, Line Guénette, Rhonda Kirkwood, Michael E. Green

**Affiliations:** 1 Université de Sherbrooke, Sherbrooke, Canada; 2 Dalhousie University, Halifax, Canada; 3 St. Francis Xavier University, Antigonish, Canada; 4 Nova Scotia Health Authority, Halifax, Canada; 5 Doctors Nova Scotia, Dartmouth, Canada; 6 McGill University, Montreal, Canada; 7 Université de Montréal, Montreal, Canada; 8 Université Laval, Québec, Canada; 9 University of Toronto, Toronto, Canada; 10 Western University, London, Canada; 11 College of Physicians and Surgeons of Nova Scotia, Bedford, Canada; 12 Queen’s University, Kingston, Canada

**Keywords:** COVID-19, Primary care, Access, Organizational innovation, Qualitative case study, Logic models, Healthcare policy

## Abstract

**Background:**

COVID-19 catalyzed a rapid and substantial reorganization of primary care, accelerating the spread of existing strategies and fostering a proliferation of innovations. Access to primary care is an essential component of a healthcare system, particularly during a pandemic. We describe organizational innovations aiming to improve access to primary care and related contextual changes during the first 18 months of the COVID-19 pandemic in two Canadian provinces, Quebec and Nova Scotia.

**Methods:**

We conducted a multiple case study based on 63 semi-structured interviews (*n* = 33 in Quebec, *n* = 30 in Nova Scotia) conducted between October 2020 and May 2021 and 71 documents from both jurisdictions. We recruited a diverse range of provincial and regional stakeholders (e.g., policy-makers, decision-makers, family physicians, nurses) involved in reorganizing primary care during the COVID-19 pandemic using purposeful sampling (e.g., based on role, region). Interviews were transcribed verbatim and thematic analysis was conducted in NVivo12. Emerging results were discussed by team members to identify salient themes and organized into logic models.

**Results:**

We identified and analyzed six organizational innovations. Four of these – centralized public online booking systems, centralized access centers for unattached patients, interim primary care clinics for unattached patients, and a community connector to health and social services for older adults – pre-dated COVID-19 but were accelerated by the pandemic context. The remaining two innovations were created to specifically address pandemic-related needs: COVID-19 hotlines and COVID-dedicated primary healthcare clinics.

Innovation spread and proliferation was influenced by several factors, such as a strengthened sense of community amongst providers, decreased patient demand at the beginning of the first wave, renewed policy and provider interest in population-wide access (versus attachment of patients only), suspended performance targets (e.g., continuity ≥80%) in Quebec, modality of care delivery, modified fee codes, and greater regional flexibility to implement tailored innovations.

**Conclusion:**

COVID-19 accelerated the uptake and creation of organizational innovations to potentially improve access to primary healthcare, removing, at least temporarily, certain longstanding barriers. Many stakeholders believed this reorganization would have positive impacts on access to primary care after the pandemic. Further studies should analyze the effectiveness and sustainability of innovations adapted, developed, and implemented during the COVID-19 pandemic.

**Supplementary Information:**

The online version contains supplementary material available at 10.1186/s12913-022-08140-w.

## Introduction

Primary care is central to high performing health systems, reducing disparities in health and improving population health [1]. Access to primary care, which includes considerations of timeliness, distance, and costs of appropriate services, is therefore essential [2]. Patients with adequate high-quality primary care access have more preventive care, better chronic disease management, fewer emergency department visits and hospitalizations, increased satisfaction, and better care coordination and health outcomes. Inadequate primary care access is a major concern facing health systems worldwide and a high priority for their populations, clinicians, and policy- and decision-makers.

A recent international report measuring primary care access found that Canada ranks poorly compared to other high-income countries for many indicators. Canada ranked 10th out of 11 countries in the 2020 Commonwealth Fund survey of the proportion of the population with a regular primary care provider [3]. Across Canada, timely access to primary care also remains a major challenge [4].

Organizational innovations have the potential to improve access to primary care by adjusting care delivery or developing new services [5]. Various organizational innovations, including centralized waiting lists (CWLs) for patients unattached to a primary care provider, advanced access models, interdisciplinary teams, community health workers, expanded scopes of practice, and virtual services, have been implemented around the world with the aim of improving access to primary care [6–8].

Worldwide, the COVID-19 pandemic spurred health systems to rapidly adapt their services [9–11]. Primary care played, and continues to play, key roles in health systems’ responses to the pandemic, including reducing avoidable emergency department visits and hospitalizations, supporting testing and vaccination, and caring for convalescing COVID-19 patients or those requiring rehabilitation services [12, 13]. In addition, primary care continues to provide non-COVID-19 care and attend to pent-up demand resulting from delayed care. To address pandemic-related primary care needs, organizational innovations have been developed or adapted, including COVID-19 testing clinics, dedicated COVID-19 clinics, apps for follow-up with COVID-19 patients in the community, and virtual care options for responding to the needs of COVID-19 and non-COVID-19 patients [14].

COVID-19 catalyzed a rapid and substantial reorganization of primary care, accelerating the spread of existing strategies and fostering a proliferation of innovations [10, 11]. To our knowledge, no study has analyzed organizational innovations implemented with the goal of improving access to primary care in the context of the pandemic. The general aim of this study was to describe the organizational innovations developed or adapted during the COVID-19 pandemic’s first 18 months to improve primary care access in two provinces in Canada. The specific aims of this study were to 1) describe contextual changes during the pandemic that influenced primary care innovations; 2) describe organizational innovations to improve primary care access adapted or developed during the COVID-19 pandemic; and 3) describe participants’ views on the potential impacts of these innovations on future access to primary care after the pandemic.

## Methods

### Study setting

We studied organizational innovations in two provinces of Canada. Canada has universal healthcare systems, administered publicly by each province. Quebec and Nova Scotia are among seven provinces which have implemented CWLs for patients who are unattached to a primary care provider due to challenges with primary care access. These regions represent both provinces highly impacted by COVID-19 cases (Quebec) and provinces less impacted by COVID-19 cases (Nova Scotia).

Quebec, home to over 8.6 million people, has the second highest population among Canadian provinces. Quebec’s health and social services system has two main governance levels: 1) the Ministry of Health and Social Services that regulates, coordinates, and oversees the system province-wide and 2) integrated health and social services centres (*Centres intégrés de santé et de services sociaux*) that plan and coordinate regional health and social services in accordance with ministerial directions. Public health and primary care are managed in parallel within these two levels of governance [15]. The main organizational model for the delivery of primary care services in Quebec is the Family Medicine Group (*Groupe de médecine de famille)*: around 370 clinics composed of six or more family physicians working in collaboration with an interdisciplinary team of nurses and allied health professionals (e.g., social workers, pharmacists) [16]. Most primary care models are publicly funded, including those privately owned and managed by family physicians. Family physicians are mainly paid fee-for-service.

Quebec implemented formal attachment to family physicians, meaning that patients are officially enrolled with a family physician who agrees to be their regular provider. Family physicians across all models of primary care (Family Medicine Groups, solo practices, community health centers) are incentivized to attach patients and to provide continuity of care to their attached patients. Most primary care clinics deliver services exclusively to their attached patients. Access to primary care remains limited for patients unattached to a family physician. Network Family Medicine Groups (*Groupe de médecine de famille – Réseau,* commonly known as super clinics) offer walk-in services to unattached patients, but substantial access gaps remain, especially outside urban areas. In 2019, 21.5% of Quebec’s population was reportedly unattached to a primary care provider. CWLs have been implemented across the province to help unattached patients find a family physician, with about 800,000 patients waiting for attachment in November 2021. Attachment remains challenging, and wait times for attachment can be well over a year.

Nova Scotia has a population of almost 1 million people, the highest of the Maritime provinces, and one of the oldest demographics in Canada. In Nova Scotia, there are two key programs funded and directed by the provincial Department of Health and Wellness (DHW): 1) IWK Health (formerly the Izaak Walton Killam Health Centre) serves children, youth, women, and families, delivering secondary and tertiary care and services, and 2) Nova Scotia Health manages primary and public care. In Nova Scotia, the majority of primary care providers are family physicians working in fee-for-service models; however, the number of family physicians remunerated via alternative payment plans (APP) has increased by 39% over the last 5 years. Over the last decade, the province has incrementally invested in collaborative family practice teams consisting of family physicians, nurse practitioners, registered nurses, and other allied health professionals. As of October 1, 2021, there were 92 collaborative family practice teams in Nova Scotia, ranging from smaller teams of at least three health professionals (with a minimum of two different professional disciplines) up to larger multidisciplinary teams composed of a larger number of health professionals from a variety of disciplines, including family physicians, nurse practitioners, dietitians, pharmacists, and social workers.

Although Nova Scotia does not have formal attachment to providers through enrollment or rostering, family physicians must adhere to standards of practice when taking on new patients. Physicians should accept patients into their practice on a first-come, first-served basis [17] and must not discriminate against patients according to the Nova Scotia Human Rights Act. Family physicians have been offered financial incentives for attaching patients to their practice and providing ongoing care [18]. In Nova Scotia, 14.4% of the population was reported as unattached as of 2019, and there has been growth in the unattached population in the province over the course of the pandemic. Over 77,000 individuals were registered on the provincial CWL (Need a Family Practice Registry) at the end of May 2021.

### Study design

The purpose of this study is to describe the organizational innovations to improve primary care access developed or adapted in the Canadian provinces of Quebec and Nova Scotia during the first 18 months of the COVID-19 pandemic. We conducted multiple case studies to describe a contemporary phenomenon – the reorganization of access to primary care – within the real-life context of the first and a half year of the COVID-19 pandemic [19]. Organizational innovations were identified by experts on our research team as well as by exchanges with key stakeholders through interviews according to a snowball strategy. We included innovations that a) aimed to improve primary care access, b) were adapted or developed during the COVID-19 pandemic, and c) changed how primary care is organized or delivered beyond a single clinic.

This study is part of the multi-provincial Canadian study, “Problems Coordinating and Accessing Primary Care for Attached and Unattached Patients Exacerbated During the COVID-19 Pandemic Year” (PUPPY Study) [20]. The overall aim of the PUPPY Study is to understand the impact of COVID-19 on access to primary care.

### Data collection

Data were collected via 1) semi-structured interviews with various stakeholders and 2) key documents related to primary care reorganization.

Participants for semi-structured interviews included provincial and regional stakeholders (policy-makers, decision-makers) and primary healthcare providers (family physicians, nurses) involved in reorganizing primary care during the COVID-19 pandemic. Using purposeful sampling [21], we ensured respondent profiles represented different roles (providers, policy-makers, and decision-makers), health system levels (local, regional, and provincial), and regions (urban, rural). Potential participants were identified by knowledge users on the research team, through the research team’s network of primary care stakeholders, and by participants. Potential participants were sent an email explaining the objectives of the study and asked to respond by email to confirm their interest in participating in an interview. Recruitment continued until saturation was reached, i.e., more interviews would not provide new ideas [22]. Thirty-three participants were from Quebec, including 15 physicians, 2 nurses, 9 stakeholders, and 7 participants with a dual role of physician or nurse/stakeholder. In Nova Scotia, a total of 30 participants were interviewed, including 20 family physicians, 9 stakeholders, and one dual-role physician/stakeholder.

Interviews were conducted online via Zoom or by telephone between October 2020 and May 2021, were digitally recorded, and lasted 45–90 minutes. MB and two research associates (MAS and VD) conducted the interviews in Quebec in French or English according to the participant’s preference. In Nova Scotia, interviews were conducted in English by three research associates (CA, LM, SN). Notes were taken and transcribed in a logbook allowing for comparisons of salient points observed during the interviews. One way we have been reflexive in this study is through regular team discussions about our interpretations of the results. Interviews were transcribed verbatim, and personally identifying information was removed. Free and informed consent was obtained prior to each interview in accordance with Research Ethics Board requirements.

The interview guide approached pre-COVID and COVID-19 periods separately, the objective being to better understand the role of the pandemic in the reorganization of access to primary care (see Additional file 1). After discussing each participant’s role, the following topics were discussed: 1) access to primary care for unattached patients and strategies to foster attachment; 2) how COVID-19 transformed access to primary care services; 3) innovations developed or adapted during the pandemic; 4) how the pandemic context fostered or hindered primary care innovations; and 5) recommendations and lessons for the future of primary care.

For key documents, we searched relevant websites (e.g., Ministry of Health, public health, health professional associations and colleges, regional health authorities) and monitored news articles related to primary care reorganization during the COVID-19 pandemic. We included publicly available documents that facilitated understanding of the primary care context during the pandemic and/or specific organizational innovations. Thirty-six documents were selected for inclusion in Quebec, and 35 documents were selected for inclusion in Nova Scotia.

### Data analysis

**Fig. 1:**
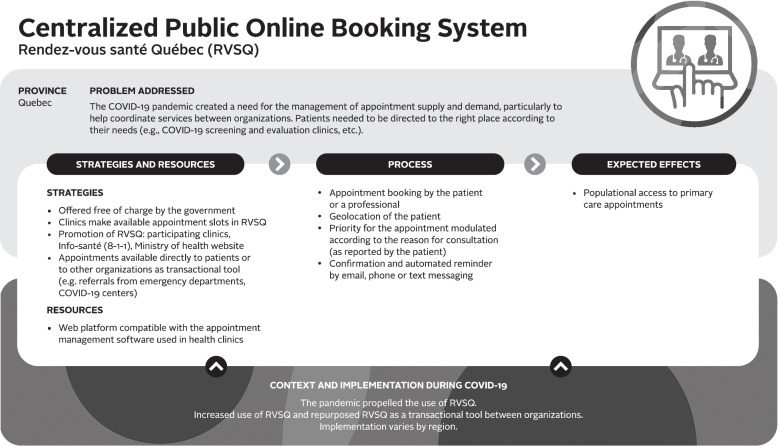
Logic model of a centralized public online booking system

**Fig. 2:**
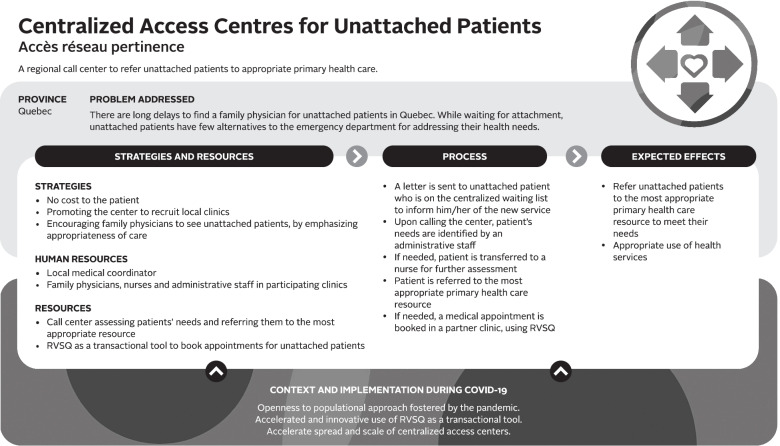
Logic model of centralized access centers to care for unattached patients

**Fig. 3:**
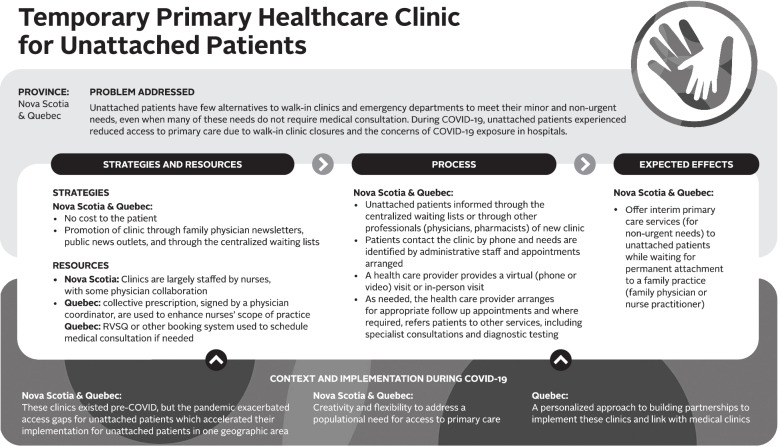
Logic model of temporary primary care clinic for unattached patients

**Fig. 4:**
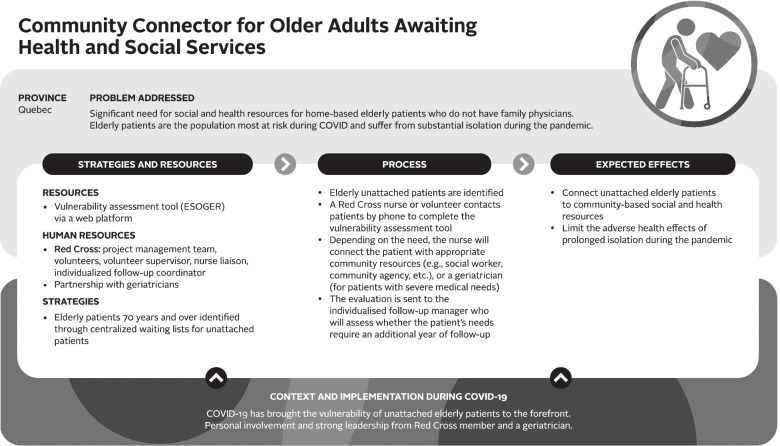
Logic model of Community Connector for older adults

**Fig. 5:**
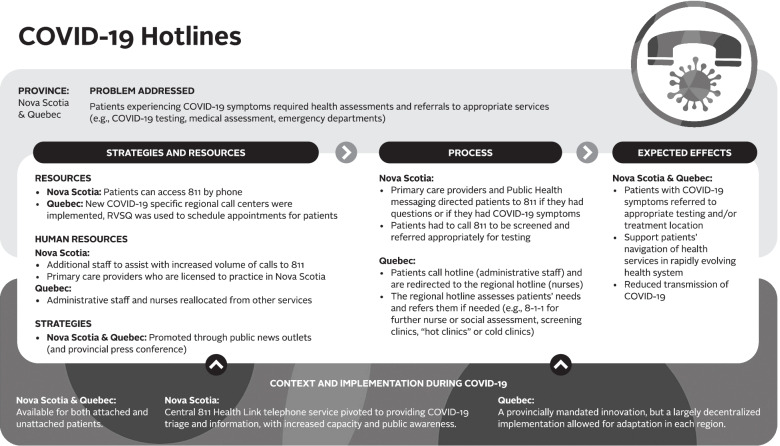
Logic model of COVID-19 hotlines

**Fig. 6:**
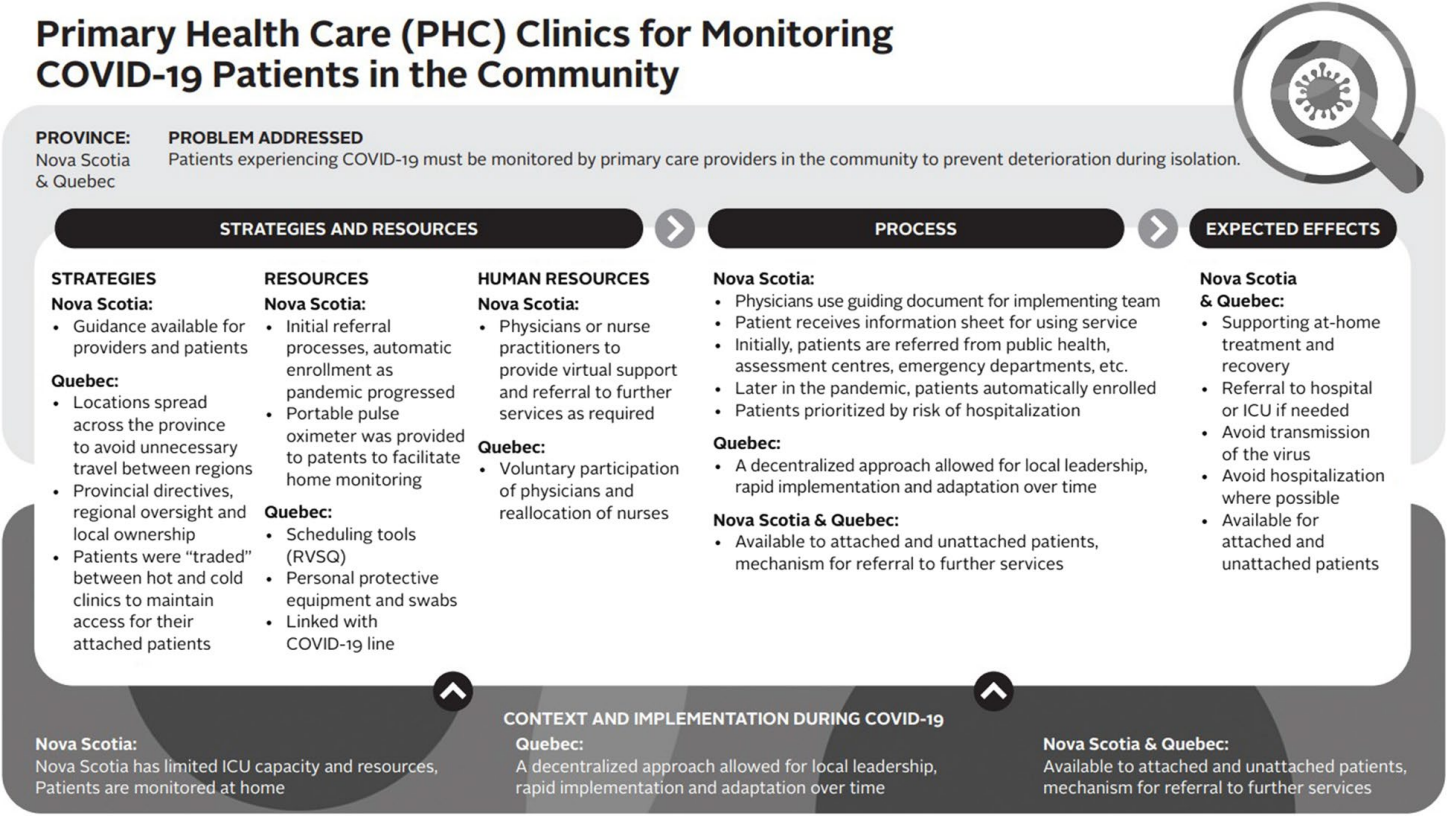
Primary healthcare clinics for monitoring COVID-19 patients in the community

Logic models were used to analyze the data – a commonly used technique for case studies. A logic model graphically depicts how a program (or innovation) works under contextual conditions to address an identified problem or need through logical sequences of inputs, processes, and intended outcomes [23]. Logic models are useful analytic tools for summarizing and integrating data from various sources. We used a logic model template based on Mitchell and Lewis’ Manual to Guide the Development of Local Evaluation Plans [24]. This particular logic model involves a diagram of main intervention components and has been used in primary care research in Canada. Table 1 presents a summary of the key components of the logic model adapted for our research purpose. Similar components are shown in logic model graphics (Figs. 1, 2, 3, 4, 5 and 6).

**Table 1:** Logic model components adapted from Mitchell & Lewis (2006)

Component	Description
Problem addressed	The broad focus of the intervention
Strategies and resources	Resources and activities needed for the intervention
Processes	Service and service system characteristics that are considered necessary to bring about lasting impacts on target individuals, communities, and/or service systems
Expected effects	Changes anticipated for individuals, communities, and/or service systems because of the intervention and measured by, for example, performance indicators
Context and implementation	Contextual elements that have guided the implementation of the intervention

We conducted thematic analysis based on an iterative mixed inductive and deductive approach [25]. Analysis of both interviews and documents was performed using NVivo12 software. Detailed summaries of each organizational innovation were prepared through an iterative process, deductively coding to logic model components, and conducting further interviews to confirm details. As the analysis progressed, several codes and categories were added to reflect the data content. The interpretation of the content was carried out through regular research team discussions.

## Results

This section first presents the contextual changes that, according to our participants, have contributed to a climate favourable to the implementation of innovations promoting access to primary care. Next, descriptions of the innovations – distinguishing between those created during the first year and a half of the pandemic and those that have been adapted to this new context – are presented in the form of logic models. Finally, we present the participants’ visions for post-pandemic organization of healthcare services (although the pandemic is still ongoing).

### COVID-19 contextual changes influencing primary care innovations

Most stakeholders described several COVID-19-related contextual changes that drastically facilitated the development or adaptation of organizational innovations to improve access to primary care. COVID-19 created an unprecedented sense of urgency and common interest to address gaps in access to primary care amongst providers, stakeholders, and patients. Specifically, the COVID-19 pandemic created a need for rapid responses to barriers in primary care access, alternatives to in-person care, and alternatives to visit modalities that were only available to attached patients. Providers’ (including family physicians’) renewed sense of community and duty was also thought to have contributed to creating a window for organizational innovations. These perceptions of key stakeholders were similar in the two jurisdictions studied. Stakeholders highlighted how this engagement in finding creative solutions contrasted with a more closed stance prior to the pandemic:

*“Considerations of infection prevention, having a population-based approach, so we took advantage of all these cracks. I think we took advantage of this momentum of flexibility, you know, or of urgency which brought a certain flexibility”* (family physician/stakeholder-QC#1).

Stakeholders in both Nova Scotia and Quebec repeatedly identified the rapid acceptance and implementation of virtual care, a previously underutilized modality, as an enormous enabler of access and opportunity for innovation:

*“You know, there's nothing like a good crisis for innovation. We had talked and talked and talked and talked about the importance of virtual care models [pre-COVID-19], and how we’d do that, and how it would impact access. And we had … some ability to do virtual care, but it was mired in so much bureaucracy and so much billing controls that nobody used it. So it was very low utility. And all of a sudden, in the space of 48 hours, we just had to do it. And so you look back at that with some pride that you completely … transformed how primary care is delivered in this province in the space of a very short time.”* (family physician-NS#13).

In Quebec, one notable change during the beginning of the pandemic was that family physicians were more willing than before to provide services to unattached patients. Providers’ openness to see unattached patients was due to a substantial decrease in overall patient demand for primary care services.

*“If we go back to spring, there was such a vacuum in the GMFs [Family Medicine Groups], people didn't go out anymore, and this created some empty walk-ins. This raised the possibility that doctors could see people who were not registered with their clientele.”* (family physician-QC#4).

Also, according to respondents, COVID-19 had a positive impact on the bureaucracy that exists in healthcare organizations by eliminating barriers and facilitating the primary healthcare provider community working together to get things done without bureaucratic impediments. Decentralized leadership (i.e., decisions were made at a more local level, for example, by local medical coordinators or physicians in management positions), particularly medical and regional leadership, as well as regional leeway to adapt to local needs were seen as having facilitated the rapid and agile response to emerging access needs during the pandemic, to both accelerate the spread of existing innovations and the creation of innovations tailored to meet local access needs.

*“There was an emergency. We came back to our value, our, our, our duty, it's not a word that we say, that we don't like to say, but to our duty as caregivers which is to care because there was an emergency situation. So, so much the better, it put us in an emergency situation and then in a mode of creativity rather than in a mode of closure.”* (family physician-QC#3)

### Organizational innovations aiming to improve access to primary care

Among the six organizational innovations aiming to improve access to primary care documented in this study using logic models, four existed prior to the pandemic but saw increased uptake and spread in the context of the pandemic, whereas the remaining two were created during the pandemic. Details are provided regarding the type of patient (attached, unattached, or awaiting attachment on the waiting list) concerned with each innovation. Figures 1, 2, 3, 4, 5 and 6 were developed using both the content of the interviews and documents analyzed, and all quotes stem from participant interviews.

#### Organizational innovations existing prior to COVID-19

##### Centralized public online booking system

In Quebec, Rendez-vous santé Québec (RVSQ) is a centralized public system for making online appointments with family physicians that existed in Quebec pre-pandemic (see Fig. 1). At first, RVSQ was intended for patients (attached, awaiting attachment, and unattached) to book medical appointments with a primary healthcare provider. This web platform was designed to be compatible with appointment management software within clinics’ electronic medical records. Patients could use RVSQ to book an appointment with their family physician, another family physician in the same clinic, or another clinic in their area, based on geographic location and availability and needs.

Before the pandemic, RVSQ had faced challenges in uptake by medical clinics across the province, and its implementation varied widely between regions. Few medical clinics across Quebec had used RVSQ prior to the pandemic. Only one region, which had proactively promoted RVSQ and supported clinics in its implementation, had seen high uptake, while implementation remained limited in other regions.

The COVID-19 pandemic created a need for the management of appointment supply and demand, particularly to help coordinate services between organizations. Emerging needs included requests for consultations in COVID-19 screening and evaluation clinics (see below for more details), redirecting symptomatic and asymptomatic patients to appropriate services, and reorienting non-urgent patients from emergency departments to primary care clinics. The pandemic transformed RVSQ into a transactional tool for providers to help coordinate services between multiple health organizations. “*RVSQ has developed a lot because we needed a transactional tool to schedule appointments quickly”* (stakeholder-QC#4). Some appointment slots were reserved and only available for providers to book an appointment based on their assessment of patients’ needs.

The main intended impact of this organizational innovation was to provide population-based access to primary care appointments. This was already the case before the pandemic, with this tool freely available to all patients to book an appointment in participating clinics, but even more so during COVID-19 given its even greater use by health professionals to coordinate services between organizations and to orient patients to the right place. Note that this innovation has not been implemented in Nova Scotia.

##### Centralized access centers to care for unattached patients

In Quebec, this innovation was born from unattached patients’ need for support in navigating the health system and for access to primary care (see Fig. 2). Based on document analysis, Quebec faces substantial gaps in the population’s access to primary care, and unattached patients have few options other than the emergency department, particularly in rural settings. This innovation included assessing the needs of patients awaiting attachment (on the waiting list) and oriented them to the most appropriate service in the community.

Access barriers for unattached patients were compounded by two primary care features in Quebec: formal attachment of patients to family physicians and the provincial continuity target for physicians to see their attached patients for ≥80% of their visits. Attachment and continuity targets were seen as hindering access for unattached patients, as they encouraged physicians and clinics to see only their attached patients: “*They are incompatible*” and limit contact between unattached patients and primary care providers. “*The doctor-unattached patient relationship had disappeared over time* (...). *We wanted to re-establish this relationship”* (family physician and stakeholder-QC#3).

To address these access gaps, a local medical coordinator led piloting and implementation, in a rural region first*,* of the *Centralized access center care for unattached patients,* creating partnerships with eight local Family Medicine Groups and services such as community pharmacy and physiotherapy clinics who agreed to provide services to unattached patients. The access center care relies on a strategy of appropriateness management, facilitated by the implementation of a call center that allows unattached patients to be guided and referred to the most relevant primary care service to meet their need. Following a needs assessment conducted by phone by a secretary or nurse, the patient is either referred to a health resource in the community or booked an appointment with a family physician. RVSQ is used as an online transactional tool to book medical appointments that are visible only to professionals from the call center.

This innovation had been piloted since 2020, and had garnered interest from the Ministry of Health and Social Services and other regions prior to the pandemic. However, COVID-19 was said to have accelerated the spread and scale-up of this innovation across the province, given that patient demand decreased, making more appointments available for unattached patients in clinics: *“We had plateaued, then COVID hit, then it was as if the project became an elegant way to put unattached patients in contact with a medical service, then there was like, I don’t know, it was like a revelation [ …*] *we were asked to deploy the project throughout Quebec”* (physician/stakeholder #3-QC). One key factor emphasized by stakeholders as contributing to the rapid spread of this innovation was local ownership and medical leadership to adapt and implement the innovation: “*Change management can never be systemic. It must always be local”* (family physician/stakeholder-QC#3). There was no analogous innovation in Nova Scotia.

##### Temporary primary healthcare clinic for unattached patients

Temporary primary healthcare clinics for unattached patients (see Fig. 3) are an innovation implemented in both Quebec and Nova Scotia to help meet unattached patients’ non-urgent needs. Across Nova Scotia, there are eight “Primary Care Clinics,” available exclusively to patients registered on the CWL (Need a Family Practice Registry)*.* These clinics provide temporary, short-term access to care while patients wait for attachment to a primary care provider.

In Nova Scotia, during the first wave of the pandemic, additional clinics in one geographical area were established or expanded to provide additional primary care access options for unattached patients and prevent them from “falling through the cracks”:

*“ … the changes we made in terms of … increased service offerings and opening up some additional primary care clinic options, I think there's been definitely positive feedback. I think we've really seen kind of the all-hands-on-deck approach in a lot of communities where people are kind of stepping up to help out. And recognizing that we don't want anyone to kind of fall through the cracks, especially during this time. Which, you know, certainly can happen for unattached patients.”* (stakeholder-NS#2)

In Quebec, there is one small nurse-led clinic in one region offering services only to unattached patients registered on the CWL. This innovation was designed and implemented by the local medical coordinator of the CWL. “*I was scandalized that, for years, we don’t offer care to this population (unattached patients)*” (family physician/stakeholder-QC#1).

Local stakeholders in Quebec emphasized that the strength of the nurse-led clinic was delivering care by nurses and referring patients to the right service if needed. As a secondary impact, the local leaders hoped that putting unattached patients in touch with family physicians to meet their one-time needs would help facilitate long-term attachment (e.g., a family physician who had seen the same diabetic patients several times upon referral through the nurse-led clinic may be more inclined to attach that patient). The nurse-led clinics are run through a collaborative effort between an administrative assistant (who takes the message), a nurse (who assesses patients’ needs), and a physician (who supports the nurse, notably with collective prescriptions, and coordinates with other primary care services). Most of the services are offered to patients by telephone. If necessary, the nurse can redirect the patient to the appropriate primary care service (e.g., in-person nurse visit, medical consultation with a family physician, community pharmacist for medication renewal or adjustment). If appropriate, the nurse can book an appointment with a family physician in a local Family Medicine Group through the online booking system (RVSQ).

While this innovation had been in development before the COVID-19 pandemic, local stakeholders in Quebec perceived COVID-19 as having facilitated the implementation of the nurse-led clinic:

*“We returned to our values, our duty [ …] as caregivers which is to CARE, because there was an emergency situation. So, for the better, [COVID-19] put us in an emergency situation, in creativity mode rather than a stance of closure. [ …] Considerations of infection prevention, treatment of the population, having a population-based approach, so we took advantage of all these cracks”* (family physician/stakeholder-QC#1).

According to the local medical coordinator and the nurse involved in this innovation, the fact that it was implemented in a small community contributed to its success.

##### Community connector for older adults awaiting health and social services

Existing prior to the pandemic, Community Connector is a Canadian Red Cross program supporting isolated seniors in the community (see Fig. 4). COVID-19 transformed this intervention, including through the use of the CWL for unattached patients to identify the most vulnerable seniors (70 years and older) and the addition of a vulnerability assessment tool (First-level Socio-Geriatric Assessment in times of Social Distancing; ESOGER) to identify seniors’ physical, social, and cognitive needs and to connect them to the most relevant community resources. The main expected impact of this innovation is to provide access to community, social, and health resources to seniors without a family physician affiliation. Also, specifically in the context of COVID-19, the use of the assessment tool was intended to evaluate, using a holistic approach to wellness, homebound seniors’ risks and to limit the adverse effects of prolonged confinement:

*“We must accompany them [the most fragile older adults] and not just make calls of convenience. We have to assess them. We have to respond to their needs because they are going to be in trouble. (...) We have to target physical health, mental health, psychological stress and then cover their social needs”* (geriatrician-QC#14).

According to a project manager, the program’s willingness to facilitate connections between community organizations and seniors, without making the process more complex for the latter, should be highlighted: “*What distinguishes them a lot is the proximity accompaniment. I think that the word proximity is something that really sets us apart and brings the services to the vulnerable person*” (stakeholder-QC#9). Finally, the assessment of each senior contacted by telephone is transmitted to an individualised follow-up manager who determines whether it is relevant to continue the follow-up of certain individuals beyond 1 year, based on the severity of their social and health needs.

According to our interviewees, the bottom-up approach combined with a willingness to support individuals and community partner organizations by the Red Cross has helped make this innovation stand out. Indeed, a manager in charge of implementing the innovation explained that “*It is already a person who is in a vulnerable situation, who has difficulties, difficulties that are increasing, so the objective is not to make everything more complex, it is rather to accompany them and then the various partners. We accompany community organizations that really don’t have many resources* “ (nurse & stakeholder-QC#9). Currently, this innovation has only been implemented in one local area of Montreal. This innovation has not been implemented in Nova Scotia.

#### Organizational innovations created during the first 18 months of the COVID-19 pandemic

In addition to the aforementioned accelerated and expanded organizational innovations, two entirely new innovations were identified. These organizational innovations were created specifically to respond to COVID-19-related needs.

##### COVID-19 hotlines

During the COVID-19 pandemic, patients experiencing COVID-19 symptoms needed to be appropriately referred for testing, assessment, or emergency care. In Quebec, COVID-19-dedicated regional call centres were created during the pandemic to respond to the rapid reorganization of services during this period (see Fig. 5). These call centres are tools to support the navigation of primary care services for all patients (attached, awaiting attachment, and unattached). At the outset, they were developed to screen prioritized populations and orient patients to the right place. They quickly became complementary to the Info-Santé (8-1-1) hotline, which also provides health advice from a professional based on a phone assessment, which had a limited capacity to handle the volume of calls resulting from the pandemic and did not have the mandate to book appointments.

The intended impact of this innovation is to first promote population-based evaluation by providing screening appointments or assessment for symptomatic COVID-19 patients. Telephone triage by a nurse was also intended to contribute to better referral of patients to primary services by promoting appropriateness management. This innovation was perceived as fostering the population’s access to primary care:

*“The introduction of COVID regional call centers really, really made a change in access, in the sense that patients who were lost or unattached, and who had COVID symptoms, had an opportunity to be seen, at least to be triaged by a nurse or prior to that and then after that, to have a contact with a physician whether it was by telemedicine or in person. The advent of this telephone appointment center has, I would say, changed the situation a lot.”* (stakeholder-QC#5)

Receptionists and nurses were rapidly hired to implement this regional COVID-19 hotline. Several retirees were hired for these new functions. Patients needing information or medical consultations call the central line, which is managed by receptionists who redirect calls to nurses answering the regional lines. When a medical appointment is required in Quebec, RVSQ is the preferred transactional tool. The regional centre directs patients according to their needs and geographic proximity.

According to many stakeholders, although this innovation was deployed in an emergency with limited resources, the rapid mobilization of health professionals and the decentralized approach to its implementation contributed to its smooth operation. The capacity improved over time with more dedicated staff.

In Nova Scotia, there was no creation of a dedicated COVID-19 hotline. Patients with COVID-related questions were invited to call HealthLink 811 which is a 24-hour, 7-days-a-week provincial telecare service. HealthLink 811 is available for all Nova Scotian patients and is staffed by nurses who provide health advice and information. 811 is also the central number for patients who wish to register for the provincial CWL via telephone.

During the COVID-19 pandemic, 811 was the central hub in Nova Scotia for COVID-19 information and screening. Patients were also asked to call 811 if they were experiencing COVID-19 symptoms so they could be screened and referred to specific services, such as dedicated COVID-19 primary care clinics or the emergency department. During the COVID-19 pandemic, additional nursing staff were hired to help with the high number of calls to 811. Due to the constant evolution of COVID-19 information and associated frequent changes to screening protocols, having a central source of reliable information was valuable.

*“The 811 line, like having that for patients with COVID questions, that was a huge support. Because it takes volume off of the front desk staff of patients calling our front desk staff, who are not clinical, and saying, “I had coughed three days ago. Like what do I do?” And they didn't have to feel pressure to answer the patient or make a recommendation. They could just say, “Oh, like call 811.” And that was really helpful.”* (family physician-NS#17)

In Nova Scotia, one stakeholder felt that the 811 line was a “one-stop shop” for unattached patients who could access the number for both COVID-19 information and to register on the CWL:

*“I think attaching [the centralized waitlist] with 811 has been an enabler because it is kind of a commonly known number. You know, people remember it, and now even more so than ever, that it’s linked to COVID screening. It definitely makes that phone number kind of a one-stop shop for folks.”* (stakeholder-NS#2)

##### Primary healthcare clinics for monitoring COVID-19 patients in the community

In both jurisdictions under study, dedicated primary care was implemented to deliver care to patients (attached, awaiting attachment, and unattached) who tested positive for COVID-19. Patients with COVID-19 were monitored by primary care providers for adverse reactions while patients isolated, thereby avoiding contagion between symptomatic and non-symptomatic COVID-19 patients (see Fig. 6). In Quebec, clinics were called “hot clinics” and were distinct from “cold” clinics, which exclusively provided services to non-symptomatic patients. In Quebec, during the first year, services for COVID-19 in dedicated primary healthcare clinics were delivered in person. These temporary COVID-19 clinics closed in the fall of 2021. In Nova Scotia, this program, referred to as COVID Community Virtual Care Team (CCVCT), is delivered virtually and still exists.

In Quebec, implementation was guided by regional directives and local appropriation according to the needs and resources of communities. “*There were no one size fits all model,”* explained a physician involved in the implementation. According to one stakeholder, this way of offering medical consultations was successful in overcoming protection material shortages:

*“I think that the model is good because in fact it allows us to separate the hot clientele [COVID-19] from the cold clientele, so I think that we, especially in our medical clinics which are not necessarily equipped to deal with all of them, and we have seen this”* (stakeholder-QC#5).

In Quebec, even though there were common practices across the province (e.g., RVSQ for appointments), daily operations varied widely between regions. There were several entry points to get an appointment at these clinics, but generally, triage was done after the patient called the COVID-19 hotline. At that point, a nurse from the regional headquarters referred the patient to a nearby hot clinic, where they would have a face-to-face consultation or teleconsultation. The participation of family physicians in those dedicated clinics was on a voluntary basis. Initially, these clinics were in-person, but moved to being virtual at the beginning of the fourth wave.

In Nova Scotia, in response to the first wave of the pandemic, the CCVCT was rolled out by Nova Scotia Health. The goal of this initiative is to support COVID-19 positive patients to manage their COVID-19 symptoms at home, thereby preventing exacerbations that may result in admissions to intensive care units or emergency departments. Initially, patients must meet eligibility criteria (i.e., known diagnosis, at risk of deterioration in the community) and are monitored virtually by physicians or nurse practitioners who are available 24/7.

In the third wave of the pandemic, the eligibility criteria and referral process were removed, and the team followed up with all positive cases over the age of 16 to identify individuals who needed monitoring.

*“So there is a COVID Community Virtual Care Team. And what it is, it's a Telus product where the person who has positive COVID, if they get discharged from hospital, then they do this assessment. So it can be done for COPD, for … Like for diseases that have like screening questions to see how well you are or if you're having an exacerbation, etc. And then it gives you instructions what to do next and where to go. Like those are things that, you know, when you see the technology and the potential for it, it's like, wow, like here's how we can have someone who can access care quickly and have information given to them so they can also be part of their self-care.”* (family physician/stakeholder-NS#7)

According to several stakeholders involved in the rollout of these clinics, rapid mobilization and the local leadership of healthcare professionals were major contributors to the success of these clinics:

*“Wow! My biggest word here is “wow” in the sense that yes, I feel the physicians are mobilized. I feel like they want to be involved. The sense of urgency, the sense of wanting to do their job. I know that I feel they are very engaged.” (*stakeholder-QC#7*)*

*“ … I would say family physicians were absolutely wonderful as a general rule in terms of stepping forward for our assessment units and for our COVID inpatient units, for even working together to provide inpatient needs and inpatient coverage.”* (family physician-NS#13)

In Nova Scotia, proactive follow-up with patients testing positive for COVID-19 allowed for timely referrals to emergency care in the case of exacerbations and provided access to primary care while patients were in isolation. This service was an important safety measure for both patients testing positive for COVID-19 and the wider community.

### Potential impacts on post-pandemic access to primary care

Although at the time of the interviews the pandemic was still ongoing, stakeholders anticipated both positive and negative impacts of these contextual changes and innovations on post-pandemic access to primary care. A few stakeholders in both jurisdictions worried that the COVID-19 pandemic would lead to increased demand for primary care after the pandemic, due to both patients postponing seeking care during COVID-19 and new needs created by the COVID-19 context (e.g., mental health needs, COVID-19 sequalae).

*“I don't agree that it's brought better accessibility. It brought a dip in demand, you know. It's like a tsunami in the background, before the tsunami the sea level decreases so if we put the demand as the level of the wave, well there in March then April, well the sea level dropped and then there slowly the wave rises.*” (family physician-QC#10)

*“We probably have missed some Paps even though we have been able to continue to do those, aside from a brief period at the beginning. You know, people are not interested in coming in to the doctor unless it’s really necessary so … the prevention piece is something that I worry about a little bit.”* (family physician-NS#8)

Some feared that pent-up demand for primary care in combination with provider fatigue and burnout caused by the burden of innovating and continuously reorganizing during the pandemic would lead to future issues in access to primary care:

*“COVID-19 is like an iceberg, it's just the part you see. Access, access problems, it's everything underneath that we don't see. The post-COVID period, you start to feel it. We're starting to feel the exhaustion of physicians. The next few months, the recovery is going to be quite challenging.”* (stakeholder-QC#8)

*“ … most of us who have been kind of on deck since March [2020] or before March, it's not really relented … I think I definitely suffered PTSD, for sure, because I was getting flashbacks of March and April [2020] … I'm seeing doctors who are at retirement place making that decision - I'm going to retire now … if we are still going into next March raging like it's raging … I think you're going to see healthcare workers drop off, and sick leaves, and all that.”* (family physician/stakeholder-NS#7)

However, most stakeholders also felt optimistic that the innovations rapidly implemented during the beginning of the pandemic would improve access to primary care in the future. A silver lining of the pandemic was that it accelerated innovation:

*“For me, it was a gas pedal. I think it's an opportunity. In the literature, change management doesn't take 2 years. It's done here and now in a short period of time, and I think that the notion of societal urgency was really one of the facilitators”* (stakeholder-QC#7).

*“So I find, you know, everything seems to be able to move a lot faster... I think from our perspective, we’ve certainly seen it be a pro. We’ve been able to kind of move things forward in a much more timely and much more responsive way that might have taken weeks, if not months in a pre-COVID kind of landscape. So that's been really positive.”* (stakeholder-NS#2)

The pandemic was said to have created a unique window of opportunity to redesign primary care and make progress on access issues that were important prior to the pandemic, but had faced substantial implementation barriers that were minimized during the pandemic. Stakeholders hoped that future access would be improved thanks to gains made during the pandemic related to virtual care (e.g., the RVSQ online appointment booking system), better navigational support for patients (e.g., regional hotlines to help patients access appropriate primary care), and population-based approaches to access (e.g., more services for unattached patients).

*“There were still improvements to be made in terms of legal recognition for pharmacists, and this was done as we went along, and with COVID-19, these are elements that are not just for pharmacists, for nurse practitioners and for other types of professionals that are very much unraveled, so this is a gain to be maintained.”* (stakeholder-QC#5)

## Discussion

Although access to primary care is central to population health, inadequate access to primary care was a major concern in Canada before COVID-19. The COVID-19 pandemic not only exacerbated the need to address primary care gaps in access, particularly for unattached patients, but has also acted as a catalyst for innovation. This study aimed to describe organizational innovations designed to improve primary care access and document related contextual changes that enabled these innovations. Building on the experience of various stakeholders from Nova Scotia and Quebec who were closely involved in the primary care transformation during the pandemic, this study identified six organizational innovations that have been developed or adapted during the first year and a half of the COVID-19 pandemic. Although these innovations are different in scope (local or regional), several common and recurring characteristics have been identified across settings, including support for patient navigation and orientation as well as services dedicated to unattached patients – with the common goal of orienting patients toward the right primary care service to meet their needs. The sense of urgency caused by the pandemic was associated with greater openness shown by both policy-makers and health professionals in the management and delivery of primary care. The adoption of teleconsultations as well as decentralized decision-making has been conducive to innovations fostering access to care, including for unattached patients and those awaiting attachment.

### Population-based responsibility to primary care and support for navigation

The pandemic context seems to have renewed interest in population-based responsibility – the mandate to maintain and improve the health and wellbeing of a geographically-defined population [26]. This contrasts with a pre-pandemic focus on clientele-based responsibility, where many providers and organizations delivered primary care mostly to attached patients, leaving unattached patients to rely on walk-in clinics or emergency departments with variable availability across the province. The Community Connector for older adults and Centralized access centers care for unattached patients highlight actions that aim to increase access for patients awaiting affiliation. Although these actions do not involve affiliation with a family physician, they provide access to primary care while patients await attachment. The need to better orient patients to care close to their home to limit travel and COVID-19 transmission reinforced the idea of creating innovations that supported navigation, thereby addressing the *accessibility* dimension of access. Online booking tools also made use of geo-localisation based on postal codes to orient patients to proximity services in their communities, thereby improving the *accessibility* of healthcare services.

Supporting patient navigation in health systems was critical to improving access in the context of the pandemic, as service delivery underwent rapid and substantial transformations. These patient navigation innovations can be mapped onto domains of access identified by Penchansky and Thomas [27] and Levesque and colleagues [2]. Navigation could provide assistance with referrals to support groups and counselling services, information on existing resources, planning appointments, completing forms and care coordination, and organizing resources to *accommodate* patient needs [14, 28–30]. Several innovations identified in our study focused on supporting patient navigation and guiding patients to *appropriate* primary care, such as regional COVID-19 hotlines, online booking platforms, and community connectors. An important component of these innovations was the use of evaluation tools to assess patients’ needs and orient them to the *appropriate* service [2].

### Repurposing existing resources in primary care and openness to changing contexts

During the pandemic period covered by our study, several primary care organizations tried transforming, through coordination and partnerships between organizations and repurposing existing resources, to provide better access to services for the population living in their region or community, whether patients were attached or unattached to a primary care provider. For example, Quebec’s online booking tool (RVSQ), developed prior to COVID-19 but with limited uptake amongst providers, was repurposed and became an important transactional tool used behind the scenes by providers and organizations to coordinate and orient patients to the right place, such as hot or cold clinics, or to reorient non-urgent patients from emergency departments to clinics near their home. Stakeholders perceived this novel use of the pre-existing booking tool as having increased uptake amongst providers and patients and as having the potential to improve access to primary care in the long term by better aligning supply with demand for primary care in local areas.

However, some innovations have changed and transformed throughout the evolution of the pandemic. For example, dedicated COVID-19 primary care has been closed, and increased follow-up of COVID-19 in the community has been conducted remotely by providers. This transformation has been adapted to the context and associated challenges of scarce professional resources. Innovations involving the monitoring of COVID-19 patients in the community were implemented outside of Canada as well. Belgium implemented a novel innovation during the COVID-19 pandemic involving physician reimbursements for providing medical advice via teleconsultations to patients potentially infected with COVID-19 and a “smart-patch” for remote monitoring of vital signs. France also recommended that potential COVID-19 patients utilize virtual care for COVID-19 diagnosis and monitoring and implemented daily self-assessment surveys for daily monitoring of COVID-19 patients [31].

Centralized governance allowed leaders to address issues raised by COVID-19. However, regional decision-makers played an important role in adapting innovations to local contexts. In Quebec, initiatives born from the pandemic came from the “top,” but there was greater uptake of innovations that emerged regionally or were “bottom-up.” Few of the innovations described were mandated provincially, leaving room to maneuver within the model to be implemented. In Nova Scotia, the innovations under study were mostly mandated by the Minister of Health through policy. We observed several regional variations in the implementation of the COVID-19-dedicated screening clinics. Several innovations implemented in Quebec emerged from local leaders responding to local needs, such as a nurse-led clinic for unattached patients, community navigators for seniors, and regional call centers to refer unattached patients to appropriate care. Medical leadership emerged in a context of reduced barriers for local leadership, fewer bureaucratic hurdles regarding guidelines, greater stakeholder collaboration, and openness to experiment with new ways of delivering care with fewer cost constraints. Several innovations emerged through the reorganization of the same resources, expanding roles of providers, or developing new settings. While recreating these contextual elements may not be feasible post-pandemic, learning from these decentralized approaches to governance and reallocation of resources may be useful for creating conditions favorable to innovation in the future.

### Implications for practice

These findings shed light on potential avenues for improving the organization of primary care services and its capacity to respond to patient demand. These avenues are:

- Allowing greater local rather than centralized leadership in the management of health services, which would contribute to more timely responses more in tune with population needs.
- Allocating financial and human resources to the reorganization of existing health service structures rather than creating new ones, which could allow for rapid adaptation to new needs.
- Supporting patients’ navigation of the healthcare system (through hotlines, for example), which would contribute to optimal use of health services (i.e., the right provider at the right time).

### Strengths and limitations

One strength of this study is that we elicited multiple perspectives (*n* = 63 interviews) until saturation in each of the two jurisdictions and complemented this with a policy scan based on more than 70 documents. Also, the fact that our participants were stakeholders involved in the implementation and/or development of the innovations allowed us to describe these from an insider’s perspective with the most up-to-date information. However, the six innovations under study are not exhaustive. We began by looking more closely at innovations related to unattached patients and access of the general population as reported by the respondents using a snowball technique. Thus, several innovations were not brought to our attention. Quebec’s nurse-led clinic and Community Connector for older adults awaiting health and social services are two examples of local level innovations that have not yet had a systemic effect on access to primary care. Yet, we believe they deserve to be included in our sample given their potential for scale-up in the future and as examples of the diversity of innovations that have taken place.

In addition, our sample does not include participants from regions where no innovations have been developed or patient participants. As the objective was to document innovations, an inclusion criterion for our participants was that they were involved in delivering or developing the innovation. We felt that policy-makers, managers, and health professionals were the stakeholders with the most complete behind-the-scenes knowledge to explain the innovations in which they have been involved. The PUPPY study does contain an important component involving patient perspectives on access to primary care. It will be interesting in the future to assess their perceptions, as service users, of the role of these innovations in access to care during the pandemic. This study primarily examined innovations relevant to the general population, rather than those developed specifically for more vulnerable population segments including seniors and unattached patients. The way we have documented how these innovations have improved access may, therefore, may not be as applicable for these subpopulations.

This was not a complete logic model analysis; rather this framework was employed to allow a description of the innovations that emerged from the analysis. The broad description of several innovations did not allow us to analyze each of those innovations in detail and assess their impact. It is important to remember that our data collection and analysis took place during a pandemic and while the innovations were being implemented. Thus, our view of these innovations may be enriched later, when the actors have more distance from the innovations and their effects on access. The timing of this study also explains the lack of quantitative data on the effect of each of these innovations on access. We rely on participants’ responses to suggest the increased use of various services (such as RVSQ), but this study is not an evaluation of the performance of those innovations.

### Future research

The question remains as to how the momentum spurred by the first months of the pandemic can be maintained. What can be retained from this innovation for a future crisis or for longer-term access? There is little research discussing how unattached patients experienced the COVID-19 pandemic and how the pandemic enabled organizational innovations. This study found that the pandemic enabled innovation through leadership agility, a collective sense of responsibility and flexibility, engagement of both provincial and regional leadership, and openness to change exhibited by the public. Although these enablers were initiated by the pandemic, they are not exclusive to public health emergencies and can be leveraged post-pandemic. Many participants in this study voiced the desire for changes to remain post-pandemic. Many innovations will be valuable for addressing access to primary care in a post-COVID-19 world, and stakeholders will need to be engaged in making decisions about which innovations are most valuable and how to maintain valuable innovations. Several innovations required redeployment of providers and resources; therefore, the sustainability of these innovations may be threatened when resources are allocated back to their original areas. Because there is a finite number of resources within the health system, improvements in primary care access may come at the cost of continuity and patient-centred care. Given the rapid implementation of these innovations, further evaluation will be needed to assess the effectiveness and sustainability of these innovations in terms of adequate access, continuity of care, and patient-centredness of care.

Considering that this study focused on two provinces in Canada, it would be interesting to expand the scope of this study by looking at organizational innovations that have been developed or transformed during the pandemic in other countries. Given the current challenges in Canada with providing appropriate access to care, understanding how other jurisdictions enable access to primary care may provide a broader picture of best practices and what needs to be done across Canada.

Most innovations we described aimed at improving access to primary healthcare and services, but their impacts on continuity and quality of care remain unknown. The pandemic has resulted in an increase in unmet need among patients because primary care is less aligned with quality guidelines when consultations, tests, exams, and referrals are postponed. Some participants referred to a tsunami, where delays in diagnosis and the management of chronic care will have an important impact on primary healthcare in the future. Future research is needed to better understand the impact of COVID-19 on the quality, continuity, and delivery of primary healthcare. Perspectives of different stakeholders, including policy-makers but also patients, should be taken into consideration.

## Conclusion

Our findings suggest the pandemic context renewed some providers’ and stakeholders’ priorities in improving access to primary care, strengthened their sense of community and population-based responsibility, temporarily reduced demand for most types of primary care, and allowed for greater policy flexibility and regional leeway – creating a unique window of opportunity to implement organizational innovations. Primary healthcare engaged in rapid transformation and role shifting: new patient needs related to COVID-19, providing testing in the community, treating patients with COVID-19, and managing acute, chronic, and preventive care in a primary healthcare setting [32]. The pandemic modified the organization and processes of primary care, and changes, in the form of innovations, collaborations, and improvements, were implemented, some of which may last beyond the pandemic [9].

This study explored the innovations developed or adapted during the first 18 months of the COVID-19 pandemic and discussed how contextual factors influenced these innovations. Primary care access is a challenge in Canada and worldwide. To ensure the viability of healthcare systems, significant changes must be made to improve access to timely and appropriate primary care, and the COVID-19 pandemic has further highlighted inequities in healthcare access and creative solutions for enabling access. It is important to see how innovations have been developed in the context of the pandemic and what facilitated these changes to provide evidence as to how continuous innovation can be incorporated into primary care. Many innovations developed and adapted during the pandemic were desirable advancements, aimed at improving the accessibility, accommodativeness, and appropriateness of primary care services. Some innovations were substantial and were implemented rapidly despite having previously lacked sufficient momentum. The pandemic has shown us that primary care can respond rapidly to healthcare needs when sufficient motivation and tools are available. This evidence should be used to improve primary care and to better prepare for future pandemics.

## Supplementary Information

**Additional file 1.** Interview guide, one interview guide including questions asked to policy-makers and/or health professionnals.

**Additional file 2.** Policy scan documents, list of all documents used by Nova Scotia and Quebec to document and describe innovations’ contexts.

## Data Availability

The datasets used and/or analyzed for this study are available from the corresponding author on reasonable request.

## Abbreviations

APP: Alternative payment plans
CCVCT: COVID Community Virtual Care Team
CWL: Centralized waiting list
NS: Nova Scotia
PUPPY: Problems Coordinating and Accessing Primary Care for Attached and Unattached Patients Exacerbated During the COVID-19 Pandemic Year
QC: Quebec
RVSQ: Rendez-vous santé Québec
