# Supplementary material for: COVID-19 – an opportunity to improve access to primary care through organizational innovations? A qualitative multiple case study in Quebec and Nova Scotia (Canada)
Source: BMC Health Serv Res. 2022 Jun 8;22:759. doi: 10.1186/s12913-022-08140-w (PMC9177136; doi:10.1186/s12913-022-08140-w)
Supplement: Supplementary file 1 — Additional file 1. Interview guide, one interview guide including questions asked to policy-makers and/or health professionnals. [file 12913_2022_8140_MOESM1_ESM.pdf]

Interview Guide – Policymakers & Providers (FP)

Instructions for Interviewer

Numbered questions (1) are required
Lettered questions (a) are required sub-questions/follow-up questions (unless already addressed by participant in numbered questions)
Lowercase Roman numerals (i.) are optional probes or optional follow-up (only to be asked if not already answered by participant)
Content in square brackets ([ ]) includes instructions for reviewers (not to be read to participant)

Introduction

Thank you for your interest being interviewed for our study. The goals of today's interview are to understand your experiences as (both) a policymaker and/or primary care provider around patient attachment and provision of health services both before and during the COVID-19 pandemic. Specifically:

1) To learn more about your experiences providing care, including for patients who do not have regular access to a family doctor or nurse practitioner (i.e. unattached patients).
2) To learn more about policies that directly and indirectly affect patient attachment to primary care, including the [centralized waitlist in your province]. These could include documented strategies, principles, rules, or planned procedures.
3) To learn from you about the barriers and facilitators to accepting new patients into primary care practice.
4) To understand how both policies and your practice has shifted overtime, particularly with the emergence and response to COVID-19, and your thoughts on how these changes relate to patient access to primary healthcare services.
5) To learn from your expertise as to where we should look for policies relevant to patient attachment in our province to ensure comprehensiveness of our policy scan

We anticipate that this interview will take approximately one hour. However, we recognize that you may be busy at this time. We are happy to accommodate your schedule and needs, such as by dividing this interview into smaller time slots if that works better for you.

Informed Consent

Your participation in this interview is entirely voluntary. You are free to withdraw your consent to participate at any time before, during, or up to one week after the interview. If you withdraw, we will not use any information you have provided to us. You may also opt to not respond to any of the questions as we go through the interview. An honorarium will be provided in thanks for your time.

The consent form contains information about the study and how the information you provide will be used. Do you have any questions about the consent form or any of the information you received?

Questions and Recording

The interview will be recorded. What you share with us will have identifying personal information removed (e.g. name, clinic name, identifying locations, etc.) and your anonymity will be protected to the best of our ability. We may use quotes from your interview in sharing our findings, but your name will never appear in any reports, and your responses will not be presented in a way that can be traced back to you.

There may be opportunities to participate in sharing our study findings. Please let us know if you are interested in participating in future knowledge translation activities, e.g. co-presenting at a conference or to media requests. These

activities would identify you as one of the participants in the study.

Interview Questions

1. Can you tell me a bit about your role in policy and the role your organization plays in the healthcare system?
    a. ***[Only ask if not mentioned]:***
        i. Organization
        ii. How long have you worked there?
        iii. Any change in roles overtime?
        iv. Any change in roles since COVID?
        v. Which internal and external groups do you work with on developing and implementing policy? what connection does your work have to primary care access?

[Pre-COVID] *Next we are going to talk about policies and practices pre-COVID-19.* ***[Please note: the below questions may have been addressed in question 1, depending on the participant. Probing and checking will be done at the discretion of the interviewer to reduce redundant lines of inquiry].***

1. **(For provider)** Were you accepting new patients into your practice pre-COVID? Can you tell me about the choices you made then for accepting patients into your practice? *Can you share some examples with me?*
    i. What was traditionally your process for determining how and when to take on new patients? Can you share some examples with me?
    ii. What could have been done to allow you to take on more patients in general?

2. **(For provider)** What recruitment methods did you implement when looking for new patients pre-COVID? (e.g., centralized waitlist only, social media postings, referrals from colleagues, etc.) Can you share some examples with me?
    i. Have you ever worked with the centralized waitlist [in your province]? [Differences pre-post COVID?]
    ii. (If applicable) How does the centralized waitlist compare to other methods of recruiting new patients?
    iii. (If applicable) What was your experience registering for the centralized waitlist?
    iv. (If applicable) What has your experience of taking new patients off the centralized waitlist been?
    v. (If applicable) Have you had any interactions with the managers/representatives of the centralized waitlists? If so, in what regard? What are your experiences with these interactions?
    vi. (If applicable) What facilitators did you encounter when joining, or receiving patients from, the centralized waitlist or other ways of recruiting patients? What were the positive outcomes or benefits that the centralized waitlist provided to you?
    vii. (If applicable) What barriers or annoyances did you experience while joining, or receiving patients from, the centralized waitlist or other ways of recruiting patients? What made these experiences negative?
    viii. (If applicable) How has COVID changed your use of the centralized waitlist?

3. Prior to COVID-19, if you thought about attaching patients to primary care providers in this province, what key policies, strategies, or incentives would come to mind, and how did they impact attachment?
    a. Can you share some examples of how they played out?
    b. ***[Only ask if not mentioned]*** What about:
        i. Provider hiring and funding policies
        ii. PHC delivery models
        iii. Financial incentives
        iv. Policies specific to CWL operations
        v. Performance metrics/ accountability measures
        vi. Programs and innovations that help meet the needs of unattached patients and/or assist with patient attachment outside of the CWL system

vii. Regulations on providers' practice (i.e., responsibilities, how to manage challenging patients, expanded role of pharmacists, etc.)
viii. Other policies that may play moderating roles in the process of attachment via CWLs

4. What are the factors that might influence the effectiveness of the centralized waitlist? How might these policies affect these factors?
    a. Can you tell me more about how? (Prompt: creation, delivery, effectiveness, or sustainability)
    b. Do you think they led to more, or less patient attachment to primary care in our province?

5. Prior to COVID-19, what were the rules, regulations, and incentives that posed obstacles for providers in taking on new patients? Which ones made things easier?
    a. How did these create obstacles or facilitators?
    b. How about rules, regulations, and incentives that impacted the ability of patients to be able to access a primary care provider? ***[Ensure participants clarify whether they are discussing from a professional or personal lens]***

[COVID] *In the next few questions, we would like to hear more about how things have changed since COVID-19*
6. (For provider) How has COVID-19 impacted accepting new patients into your practice?
    a. Would you anticipate being able to accept new patients into your practice once the pandemic restrictions are over?

7. **(For provider)** What key changes have you experienced in your practice since the onset of COVID-19?
    i. Changes in practice, PPE use, virtual care, billing codes used, prescribing? (e.g. the roles or functions you carried out for patients with/suspected to have COVID19 and others)
    ii. Changes in the ways you communicated and interacted with patients?
    iii. Changes in the ways you communicated and interacted with other healthcare providers and services, namely pharmacists?
    iv. Ability to provide care for patients?
    v. Ability to take on new patients?
    vi. Have there been any changes in your interactions with the centralized waitlist?
    vii. Other changes?

8. **(For provider)** What was your experience implementing these changes?
    i. How were decisions about COVID-related changes made?
    ii. How were changes communicated?
    iii. What supports were available for you to carry out these roles?
    iv. What challenges did you encounter in your practice related to these changes?
    v. Were there any unintended consequences?
    a. Looking at the list of PROPOSED roles during the different stages, what supports would be needed to enable you to carry out these roles? What were the barriers?
    b. Are there other roles that family physicians could have played during the different stages? What supports would be needed to carry those out?

9. **(For provider)** What changes if any, have you noticed in the kinds of patients you are typically seeing since the COVID pandemic began? Have you noticed any changes in the needs patients have been discussing with you?

10. Have there been any key policy changes implemented since COVID-19 relevant to patients' access and attachment to primary care providers? If so, what are these?
    a. **[Optional prompts below]**
        i. Any policies that account for the needs of patients who were unattached before COVID?

ii. Any policies that address the issues faced by patients with complex chronic health conditions?
iii. How virtual care may have impacted patient access and attachment to primary care?
iv. How the reallocation of primary care staff may have impacted patient access and attachment to primary care?
v. The expanded role of pharmacists in managing both attached and unattached patients?
vi. Any unintended consequences you noted?

11. Did the process used to develop and implement COVID-19-related policies differ from what happened before COVID-19? If so, how? ***[Optional prompts below]***
    i. Planning process?
    ii. Dissemination/Communication?
    iii. Evaluation?

12. What response or feedback did you get regarding COVID-19 policy changes related to patient access and attachment to primary care?
    a. From whom did you receive that response and why do you believe they responded in that way?

13. In what ways, if any, has the centralized waitlist been impacted by the COVID-19 response?
    a. Can you tell me about the reasons for these changes?

14. What unintended consequences, if any, from these policies emerged? What was your experience with these? Can you tell me how they were identified and addressed?

[Post-COVID] *The last section of questions will look beyond the peak of the COVID-19 pandemic [Prospective]*

15. Are there any COVID-19-related policies or practice changes since COVID that you think we should keep following the pandemic? If so, which ones (e.g. virtual care)?
    a. Why do you think that?

16. Are there any practice changes or COVID-19-related policies relevant to patient access and attachment to a primary care provider that you think we should reverse/change following the pandemic? If so, which ones?
    a. What about these policies makes you want to reverse them in the longer term?

17. One of our goals today is to identify documents that we should be including in our policy scan. What words would you use to talk about these policies? What are the key words you use in communications around these policies? Which words might appear in key documents that we should look for? What documents or sources should we include?

18. Are there any "lessons learned" that you would like to share with us?

19. Anything else you would like to add?

<u>Closing</u>

Thank you so much for your time in speaking with me today. May we contact you if we have any clarifying or follow up questions?

❑ Yes
❑ No

What is your preferred method to be contacted?

E-mail: ______________________________

Phone: ______________________________

Other: ______________________________

Our team will be completing other interviews and will be writing preliminary reports and the final analyses. Would you like us to share both of those with you? Would you like to contribute to the analysis and/or interpretation of the de-identified summaries of these data?

- ❑ Yes
- ❑ No

Interview completed on (YYYY-MM-DD): ______________________

Interview length (HH:MM):____________________
