# Supplementary material for: COVID-19 – an opportunity to improve access to primary care through organizational innovations? A qualitative multiple case study in Quebec and Nova Scotia (Canada)
Source: BMC Health Serv Res. 2022 Jun 8;22:759. doi: 10.1186/s12913-022-08140-w (PMC9177136; doi:10.1186/s12913-022-08140-w)
Supplement: Supplementary file 2 — Additional file 2. Policy scan documents, list of all documents used by Nova Scotia and Quebec to document and describe innovations’ contexts. [file 12913_2022_8140_MOESM2_ESM.pdf]

Policy scan documents Nova Scotia and Quebec

**Nova Scotia**	
**Name**	**Hyperlink**
HealthLink 811 Line Launched	https://novascotia.ca/news/smr/2009-07-29-healthlink.asp
Physician's Bulletin - Fees and Billing Reminders	http://msi.medavie.bluecross.ca/wp-content/uploads/sites/3/2018/07/MSI-Physicians-Bulletin-July-2018.pdf
Coronavirus (COVID-19): vaccine	http://web.archive.org/web/20210514202101/https://novascotia.ca/coronavirus/vaccine/
COVID-19 Affected Areas Expanded	https://novascotia.ca/news/release/?id=20200228006
Nova Scotia College of Nursing COVID-19 Info & Resources	https://app.webtrackz.com/?q=email/view/14WnhzuxjXSXUO0T9RrdVKbqROs9IBI6T6x1_vOPUsOcZlqjNX4QtYJQ
NSHA has opened initial COVID-19 assessment centres across the province	https://www.nshealth.ca/news/nsha-has-opened-initial-covid-19-assessment-centres-across-province
Virtual Appointment Practice Guidelines - March 16, 2020	https://files.constantcontact.com/bb5ecdfa601/142e8fdd-134d-49c2-9f52-9123e6e9ccd2.pdf
Department of Family Practice	https://myemail.constantcontact.com/COVID-19-Family-Practice-Update-March-17--2020.html?soid=1128231921083&aid=EP5fBGo8RHE
Physician's Bulletin - Telehealth during COVID	http://msi.medavie.bluecross.ca/wp-content/uploads/sites/3/2020/03/March-18-2020-Bulletin-COVID-19.pdf
Dispensed Days Supply; Prescribing in a Public Health Emergency/Crisis	https://www.nspharmacists.ca/wp-content/uploads/2020/03/Notice_DaySupplyPrescribingHealthEmergency.pdf
SUBSECTION 56(1) CLASS EXEMPTION FOR PATIENTS, PRACTITIONERS AND PHARMACISTS PRESCRIBING AND PROVIDING CONTROLLED SUBSTANCES IN CANADA DURING THE CORONAVIRUS PANDEMIC	https://cpsns.ns.ca/wp-content/uploads/2020/03/CDSA_Exemption_InterpretiveGuideForControlledSubstances_March19_2020.pdf
COVID-19: Important Information for Pharmacists and Prescribers	http://pdbns.ca/uploads/publications/COVID-19-Bulletin-03-20-2020.pdf
Restricted licences for recently retired physicians – limited to pandemic response as directed by NSHA and IWK	https://cpsns.ns.ca/restricted-licences-for-recently-retired-physicians-covid-19/

Licensing Newly Graduated, Canadian-trained, Post-Graduate Trainees during the Pandemic	https://cpsns.ns.ca/wp-content/uploads/2020/04/LicensingNewlyGraduated_PGTs_Dur-ingCOVID19_April6_2020.pdf
Department of Family Practice - EHS Field Assessment Units	https://myemail.constantcontact.com/COVID-19-Family-Practice-Update-April-9--2020.html?soid=1128231921083&aid=y2S3HrYIDV8
Call for COVID-19 Support Positions	https://www.nscn.ca/professional-practice/trending-topics/covid-19#call
Temporary Suspension of 180 Days Renewal Limit; Keeping DIS Current	https://www.nspharmacists.ca/wp-content/uploads/2020/04/Notice_RenewalsDISQueue.pdf
COVID Community Virtual Care Team	https://myemail.constantcontact.com/COVID-19-Family-Practice-Update-April-17--2020.html?soid=1128231921083&aid=-GV3GuHGOj0
COVID Community Virtual Care Team	http://r20.rs6.net/tn.jsp?f=0018bdaCM_hs5GAzMVvkg8zDx7vyiSbnNxzTgYp5h6gf102aqIFj1VtJ2kJc8bWS-BjttSuuV3dcwl1dF422fWGoy12CWb8A39sMIFnH-FzEmIC_KYZTkOzgKk3NLSU-crR5Z_5VjNb33JJ7xdMo4us6ae7RxJPiXrCVebZsY7m5cWKQRJ7WQs-hAPiktsI5ydATc2TIU87RvYY_RgG4uKcS-kUAFSfTNeB7M9-5iD5vC3D-uk5lK_Ni8bqcoVbEevIJBixfyFy7CPk5VDAyQUYSdscumGwjEhSTCTyhiDe4HWT8O9OyDoTZ1X-w==&c=luMSpRWJ-fbaw52E0E2NxnXmpvkj1rCFyblOPYxGvRXVxXsUDJw9SQ==&ch=0tQM0_VBt-abC0yC5QUNWs3V0WiUYa9GgnqsjynYGg8bG6GyXOTVGYQ==
COVID Community Virtual Care Team - Patient Information	http://r20.rs6.net/tn.jsp?f=0018bdaCM_hs5GAzMVvkg8zDx7vyiSbnNxzTgYp5h6gf102aqIFj1VtJ2kJc8bWS-BjtKu3lwTUBEkAC4ofzp4oxmwIg3fvZtQRG7H9pbnkQTQuN2Xu0cI9UV80W_7c-zE2EvXP2sLQPth__7JwB-Y6bVLn80LkhxmBlU1EJmAhHPkGqkBLlt7hvmrMnI_6-8hctWgPwHRqzrfe1Mduqo32D3-VwV24X8Yt6drXR058ZjS1cOWvcbu82WFnByJ0lStDpZS5G-9YUiKRNqdOPD2kWxTdmvf4d-p7j&c=luMSpRWJ-fbaw52E0E2NxnXmpvkj1rCFyblOPYxGvRXVxXsUDJw9SQ==&ch=0tQM0_VBt-abC0yC5QUNWs3V0WiUYa9GgnqsjynYGg8bG6GyXOTVGYQ==
Prescribing Discharge Medications during COVID19	No link
Nova Scotians no longer limited to 30-day prescriptions as dispensing rules relax	https://www.cbc.ca/news/canada/nova-scotia/pharmacies-drug-supply-covid-19-easing-1.5575393
Online Booking Available for Colchester East Hants Health Centre Blood Collection and Woodlawn Blood Collection	No link

Nova Scotia Health, IWK rolling out online booking service	http://www.nshealth.ca/news/nova-scotia-health-iwk-rolling-out-online-booking-service
Nova Scotia hires additional 811-system staff to deal with spike in COVID testing demand	https://www.saltwire.com/atlantic-canada/news/provincial/nova-scotia-hires-additional-811-system-staff-to-deal-with-spike-in-covid-testing-demand-498623/
Asymptomatic Testing for Bar Staff and Patrons	https://novascotia.ca/news/release/?id=20201124001
Mobile Testing Units Available for Community COVID-19 Testing	https://novascotia.ca/news/release/?id=20201201006
Restrictions Extended, More Asymptomatic Testing Rolls Out Across the Province	https://myemail.constantcontact.com/COVID-19-Family-Practice-Update-December-4--2020.html?soid=1128231921083&aid=jihtWAxkL6g
Coronavirus Update #136 - Monday, January 11, 2021	https://covid19hub.nshealth.ca/blog/Coronavirus-Update-136-Monday-January-11-2021
Emergency licensure available to support COVID-19 Immunization	https://cpsns.ns.ca/emergency-licensure-available-to-support-covid-19-immunization/
COVID-19 Mobile Vaccination Clinics	https://novascotia.ca/news/release/?id=20210316006
COVID Pop-up Volunteer Sign-Up	https://docs.google.com/forms/d/e/1FAIpQLSd55PY-PoEKWIpt7lEWHOBe5Y95l69A7Vn62wlKhPX6WJSQiPg/viewform
Coronavirus Update #172 - Sunday, May 2, 2021	https://covid19hub.nshealth.ca/blog/Coronavirus-Update-172-Sunday-May-2-2021
Nova Scotia Health launches VirtualCareNS Pilot	https://www.nshealth.ca/news/nova-scotia-health-launches-virtualcarens-pilot
VirtualCareNS	http://web.archive.org/web/20211105110415/https://www.nshealth.ca/content/virtualcarens
**Quebec**	
Veille sur les outils numériques en santé dans le contexte de COVID-19	https://observatoire-ia.ulaval.ca/veille-sur-les-outils-numeriques-en-sante-dans-le-contexte-de-covid-19/#:~:text=En%20rediffusion-,Veille%20sur%20les%20outils%20num%C3%A9riques%20en,le%20contexte%20de%20COVID%2D19&text=La%20pand%C3%A9mie%20de%20la%20COVID,risques%20de%20propagation%20du%20virus.

Guide de gestion relatif à l'amendement 140 Modalités fondées sur le versement d'un supplément associé à la première visite de prise en charge d'un nouveau patient orphelin par le médecin de famille	https://fmoq.s3.amazonaws.com/pratique/ententes/2015/Guide-de-gestion-Amendement-140.pdf
Lettre d'entente no 245 – Nouvelle version remplaçant celle en vigueur depuis le 1er novembre 2011	Lettre d'entente no 245 – Nouvelle version remplaçant celle en vigueur depuis le 1er novembre 2011 (gouv.qc.ca)
Rapport du vérificateur général du Québec (chapitre 5).	2015-05-27 Vérification de l'optimisation des ressources – Rapport du Vérificateur général du Québec à l'Assemblée nationale pour l'année 2015-2016, printemps 2015 (vgq.qc.ca)
Classification de priorité d'inscription au Guichet d'accès à un médecin de famille (GAMF)	No link
Cadre de référence provincial des guichets d'accès pour la clientèle sans médecin de famille	No link
Processus d'inscription dans consultation	http://fmoq.s3.amazonaws.com/pratique/organisation-de-la-pratique/guichets-acces/VisuelProcessusInscription.pdf
Cadre de référence provincial des guichets d'accès pour la clientèle sans médecin de famille (GACO)	https://www.cisss-bsl.gouv.qc.ca/sites/default/files/fichier/4.1.2_cadre_de_reference_-_mecanismes_regionaux_dacces_aux_services_de_premiere_ligne_pour_la_population_sans_medecin_de_famille_1.pdf
Programme de financement et de soutien professionnel pour les groupes de médecine de famille	https://publications.msss.gouv.qc.ca/msss/document-001527/
Programme de désignation réseau pour les groupes de médecine de famille	https://publications.msss.gouv.qc.ca/msss/document-001630/#:~:text=Le%20Programme%20de%20d%C3%A9signation%20acc%C3%A8s,qui%20en%20font%20la%20demande.
Modifications apportées aux codes et aux catégories de problèmes de santé ainsi qu'aux groupes de vulnérabilité	https://www.ramq.gouv.qc.ca/SiteCollectionDocuments/professionnels/infolettres/2017/info171-7.pdf
Lettre d'entente 321	https://www.ramq.gouv.qc.ca/SiteCollectionDocuments/professionnels/infolettres/2018/info101-8.pdf

Lettre d'entente 321. La grande inscription	https://www.ramq.gouv.qc.ca/SiteCollectionDocuments/professionnels/infolettres/2017/info233-7.pdf
Rapport du protecteur du citoyen. Améliorer le processus d'inscription auprès d'un médecin de famille.	https://protecteurducitoyen.qc.ca/sites/default/files/pdf/rapports_d_intervention/ameliorer-processus-inscription-medecin-famille.pdf
Loi modifiant l'organisation et la gouvernance du réseau de la santé et des services sociaux notamment par l'abolition des agences régionales	https://www.legisquebec.gouv.qc.ca/fr/pdf/lc/O-7.2.pdf
Cadre de gestion des groupes de médecine de famille universitaires (GMF-U)	https://publications.msss.gouv.qc.ca/msss/fichiers/2019/19-920-01W.pdf
Améliorer l'accès aux soins de santé en première ligne -Le gouvernement abolit les frais pour plusieurs services pharmaceutiques	Améliorer l'accès aux soins de santé en première ligne - Le gouvernement abolit les frais pour plusieurs services pharmaceutiques (newswire.ca)
Loi favorisant l'accès aux services de médecine de famille et de médecine spécialisée	https://www.legisquebec.gouv.qc.ca/fr/document/lc/A-2.2
Statutaire réseau GAMF / Lancement de la Communauté de pratique Guichet d'Accès à un Médecin de famille et à l'équipe Interdisciplinaire	No link
Services en ligne de la Régie – Nouveau rapport « Nombre de patients inscrits pour la tarification »	https://www.ramq.gouv.qc.ca/SiteCollectionDocuments/professionnels/infolettres/2016/info074-6.pdf
Amendement no 183 : Modifications diverses à votre entente	https://www.ramq.gouv.qc.ca/SiteCollectionDocuments/professionnels/infolettres/2019/info087-9.pdf
EP 40 – Médecine de famille, prise en charge et suivi de la clientèle	https://www.ramq.gouv.qc.ca/SiteCollectionDocuments/professionnels/manuels/syra/medecins-omnipraticiens/104-brochure-1-omnipraticiens/Omnipraticiens_Brochure_no1.html#85719
Lettre d'entente N.229	https://www.ramq.gouv.qc.ca/SiteCollectionDocuments/professionnels/manuels/syra/medecins-omnipraticiens/104-brochure-1-omnipraticiens/Omnipraticiens_Brochure_no1.html#89196

Inscription d'un lot de patients orphelins sans visite	https://www.ramq.gouv.qc.ca/fr/professionnels/medecins-omnipraticiens/facturation/inscription-clientele/Pages/inscription-lot-patients-orphelins-sans-visite.aspx#:~:text=Comme%20m%C3%A9decin%20omnipraticien%2C%20vous%20pourriez,vous%20seront%20attribu%C3%A9s%20par%20lot.
Inscription de la clientèle. Calcul du taux d'assiduité	https://www.ramq.gouv.qc.ca/fr/professionnels/medecins-omnipraticiens/facturation/inscription-clientele/Pages/calcul-du-taux.aspx#:~:text=Le%20calcul%20du%20taux%20d,inscrite%20au%20registre%20des%20consultations.
COVID-19 : regard sur la fréquentation dans les urgences au Québec	https://www.inesss.qc.ca/fileadmin/doc/INESSS/COVID-19/INESSS_Covid_Urgences_EP.pdf
COVID-19 : regard sur la fréquentation des urgences au Québec. Coup d'œil	https://www.inesss.qc.ca/fileadmin/doc/INESSS/COVID-19/CoupDoeil_Frequentation_Urgences.pdf
Accès réseaux pertinence en première ligne	No link
État d'avancement projet Accès réseau pertinence	No link
La COVID-19 cause une baisse de services dans les cliniques et les GMF	La COVID-19 cause une baisse de services dans les cliniques et les GMF | Coronavirus | Radio-Canada.ca
Directives cliniques aux professionnels et au réseau pour la COVID-19.	https://www.msss.gouv.qc.ca/professionnels/covid-19/directives-cliniques-aux-professionnels-et-au-reseau/
Ou trouver un médecin en temps de pandémie ?	https://www.latribune.ca/2020/10/09/ou-trouver-un-medecin-en-temps-de-pandemie-43efb5413eef3608528c936bcd98227e
Un million de visites en moins dans les urgences l'an dernier	https://www.lapresse.ca/actualites/sante/2021-07-01/un-million-de-visites-en-moins-dans-les-urgences-l-an-dernier.php
Téléconsultations : le Collège des médecins rappelle à l'ordre les médecins de famille	https://ici.radio-canada.ca/nouvelle/1787161/teleconsultation-medecins-famille-quebec
Les patients « orphelins » auront un accès plus aisé aux soins de santé	Les patients «orphelins» auront un accès plus aisé aux soins de santé - Le Reflet du Lac
COVID-19 : les pharmaciens du Québec veulent l'abolition des frais aux patients	https://ici.radio-canada.ca/nouvelle/1744822/coronavirus-covid-19-frais-pharmaciens-abolition-patients
